# Familial Recurrence of Cerebral Palsy with Multiple Risk Factors

**DOI:** 10.1155/2011/307857

**Published:** 2012-01-11

**Authors:** Lawrence P. Richer, Nancy A. Dower, Norma Leonard, Alicia K. J. Chan, Charlene M. T. Robertson

**Affiliations:** ^1^Division of Neurology, Department of Pediatrics, University of Alberta, 4-588 Edmonton Clinic Health Academy (ECHA), Edmonton, AB, Canada T6G 1C9; ^2^Division of Hematology, Department of Pediatrics, University of Alberta, Edmonton, AB, Canada T6G 1C9; ^3^Department of Medical Genetics, University of Alberta, Edmonton, AB, Canada T6G 2H7; ^4^Division of Neurodevelopmental Pediatrics and Rehabilitative Medicine, Department of Pediatrics, University of Alberta, Edmonton, AB, Canada T6G 0B7

## Abstract

The recurrence of cerebral palsy in the same family is uncommon. We, however, report on two families with two or more affected siblings. In both families, numerous potential risk factors were identified including environmental, obstetric, and possible maternal effects. We hypothesize that multiple risk factors may lead to the increased risk of recurrence of cerebral palsy in families. Intrinsic and maternal risk factors should be investigated in all cases of cerebral palsy to properly counsel families on the risk of recurrence. Recent studies of genetic polymorphisms associated with cerebral palsy are considered with reference to our observations in these two families.

## 1. Introduction

Cerebral palsy is a static encephalopathy and clinical syndrome of restricted movement and posture with numerous possible etiologies. Prenatal, perinatal, and postnatal factors may contribute to the development of cerebral palsy (CP) and vary based on the gestational age. Perinatal factors such as placental abruption or uterine rupture may lead to the development of CP in the child. Other factors including premature birth, intrauterine infection, ischemic stroke, congenital malformations, restricted or excessive intrauterine growth, and complications of multiple gestations or maternal fever in labor are also associated with CP [1]. While our understanding of the CP is improved, frequently a specific etiology cannot be identified. Population-based CP registries have identified numerous risk factors, but despite advances in obstetrical care and fetal monitoring, there has been no overall change in the prevalence of CP over time [2]. While the majority of families have only one child with CP, there are a few reports of recurrent CP within families [3–5]. We report on two families in whom two or more siblings have been diagnosed with CP. These cases challenge us when counseling families and underscore the need to be thorough in the evaluation of children with CP and their families.

## 2. Case Reports

Ethics approval was obtained from the University of Alberta Health Research Ethics Board, and informed consent was obtained from the parents.

### 2.1. Family 1

A Metis mother in a nonconsanguineous marriage delivered three healthy children and then from age 26 through 29 years had three children with cerebral palsy. She had regular prenatal care and used prenatal vitamins with folate for pregnancies. She did not smoke but had some alcohol intake with the first four pregnancies. The first child of this case study, from the mother's fourth pregnancy, was a male born via vaginal birth at 41 weeks gestation complicated only by a nuchal cord. Placental pathology was not performed. His birth weight was 2713 grams (between 5th and 10th percentile), head circumference 32.5 cm (5th percentile), with Apgar scores of 5 at one minute and 7 at five minutes. Transient hypoglycemia and apnea prompted admission to the neonatal nursery where a patent ductus arteriosus was discovered. Subsequent delay in motor development, microcephaly, and poor visual behavior were noted on followup. Neurological examination at one year of age showed severe (modified Ashworth scale 3) and symmetric spastic hypertonia in the upper and lower extremities with severe language impairment and small stature. Over time, there was no developmental regression, and the clinical diagnosis of spastic quadriplegic cerebral palsy was made. Magnetic resonance imaging (MRI) demonstrated ventriculomegaly, white matter hypoplasia, hypoplasia of the corpus callosum, and signal abnormalities within the periventricular white matter and globus pallidus.

During mother's fifth pregnancy, polyhydramnios at 30 weeks gestation led to a diagnosis of dextrocardia, single atrioventricular valve, and unobstructed double-outlet right ventricle in the fetus. Prenatal MRI at 34 weeks gestation demonstrated a left frontal hemorrhagic infarction. This boy was born at 36 weeks 5 days gestation following induction with a birthweight of 2770 grams (80th percentile), head circumference of 32.5 cm (50th percentile), and Apgar scores of 8 at one and five minutes. Placental pathology demonstrated a small intervillous hematoma, and the feto : placental weight ratio was low at 6 : 3, but there was no histological evidence for chorioamnionitis. Chromosomal analysis and testing for a 22q11 deletion were normal. An MRI at 10 days of age showed multiple subacute and chronic areas of parenchymal hemorrhagic infarction within the frontal lobes, left occipital lobe, and left cerebellum as well as a right porencephalic cyst, hypoplastic corpus callosum, and delay in myelination. Over time, severe spastic hypertonia (modified Ashworth scale 3) developed symmetrically in both the upper and lower extremities in keeping with the clinical diagnosis of spastic quadriplegic cerebral palsy. In addition, the child had a seizure disorder, cortical visual impairment, microcephaly, and severe language impairment. The child subsequently died at 1.5 years of age from complications of his complex congenital heart disease.

During the mother's sixth and last pregnancy, she noticed weakness of her right arm and face with slight right leg weakness and slurred speech at 23 weeks 3 days gestation. Initial investigation and management were done at another site. An MRI and magnetic resonance angiogram demonstrated a left frontal lobe hemorrhagic infarction as well as confluent signal abnormalities in the periventricular white matter and posterior limb of the internal capsule. While carotid ultrasounds were normal, a transesophageal echocardiogram showed a patent foramen ovale. Heparin was initiated and used for two weeks. There has been no recurrence of stroke symptoms, but significant short-term memory loss and migraine headaches persist.

**Figure 1:**
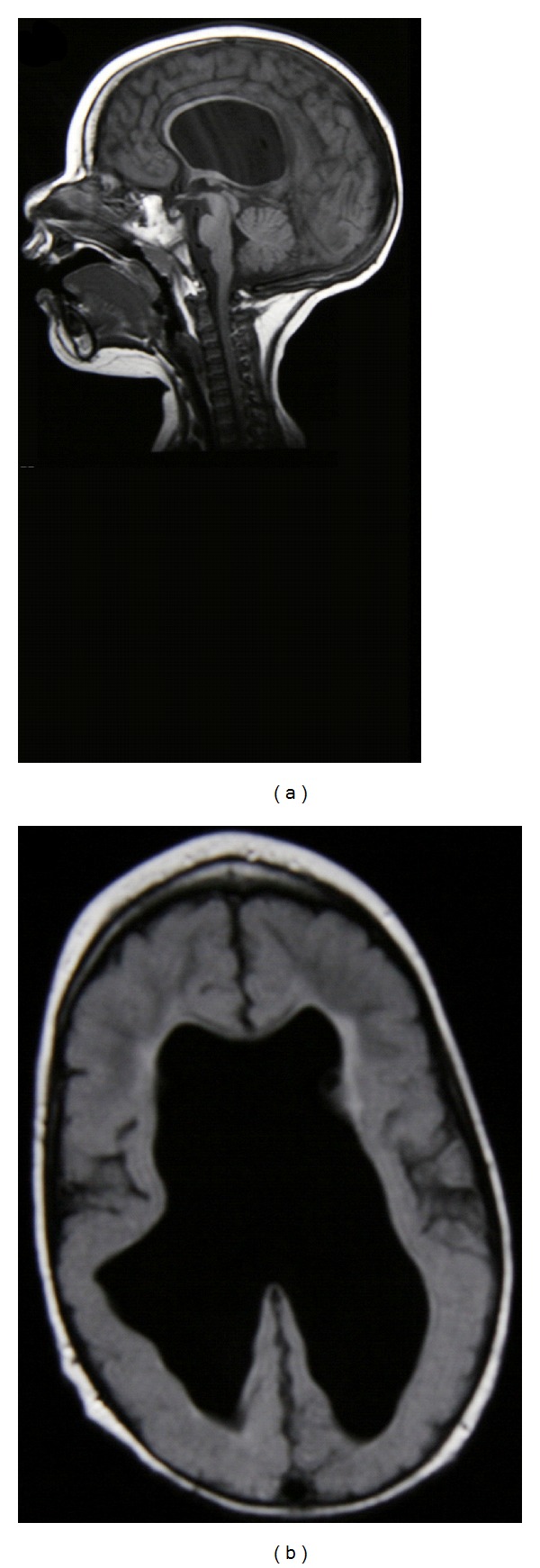
Sagittal T1 (a) and transverse FLAIR (b) MRI of sixth pregnancy (third case) in family 1. Note thin corpus callosum in (a). Irregular ventricular borders, white matter hypoplasia, ventriculomegaly, and periventricular hyperintense signal are demonstrated in (b).

A female fetus was found to have ventriculomegaly on prenatal ultrasound and was delivered by cesarean section at 36 weeks gestation. The birth weight was 2870 grams (25th percentile), head circumference 36.5 cm (75th percentile), and length 45 cm (5th percentile). Apgar scores were 3 at one minute and 9 at five minutes. Postnatal ultrasound demonstrated a right porencephalic cyst and bilateral ventriculomegaly and a ventriculoperitoneal shunt was inserted at two months of age. Followup demonstrated severe global developmental delay, cortical visual impairment, and normal hearing. Motor examination showed severe spastic hypertonia (modified Ashworth scale of 3) symmetrically in both the upper and lower extremities. MRI showed significant loss of white matter in both right and left cerebral hemispheres, with dilatation and irregularity of the lateral ventricular borders, and a thin corpus callosum (Figure 1). The child has shown no developmental regression, and follow-up MRI has remained stable in keeping with the clinical diagnosis of spastic quadriplegic cerebral palsy.

The mother was found to have slightly low levels of protein S (0.55 U/mL (0.62–1.22)) and antithrombin III (0.80 U/mL (0.87–1.29)). In addition, factor VIII levels were high at 1.98 U/mL (0.5–1.5) as were ristocetin cofactor (1.73 U/mL (0.47–1.44) and vonWillebrand factor antigen (1.96 U/mL (0.51–1.72)). Anticardiolipin antibody was “low to medium positive” at 23. The remainder of the prothrombotic workup on the mother was normal including antinuclear antigen, testing for factor V Leiden thrombophilia (activated protein C resistance assay and factor V gene DNA analysis), prothrombin gene DNA analysis, protein C, homocysteine, immunoglobulin G (IgG) and M (IgM) antibodies to cardiolipin, and phosphatidylserine. Mitochondrial DNA testing for mitochondrial encephalomyopathy, lactic acidosis, and stroke-like episodes (MELAS) was normal. Skin biopsy testing for electron-dense granules in arterioles in keeping with cerebral autosomal dominant arteriopathy with subcortical infarcts and leukoencephalopathy (CADASIL) was also negative in the mother. The same tests for thrombophilia were performed on the three children with CP, and all were normal. Screening tests for common congenital infections and inborn errors of metabolism were also normal including plasma lactate.

### 2.2. Family 2

**Figure 2:**
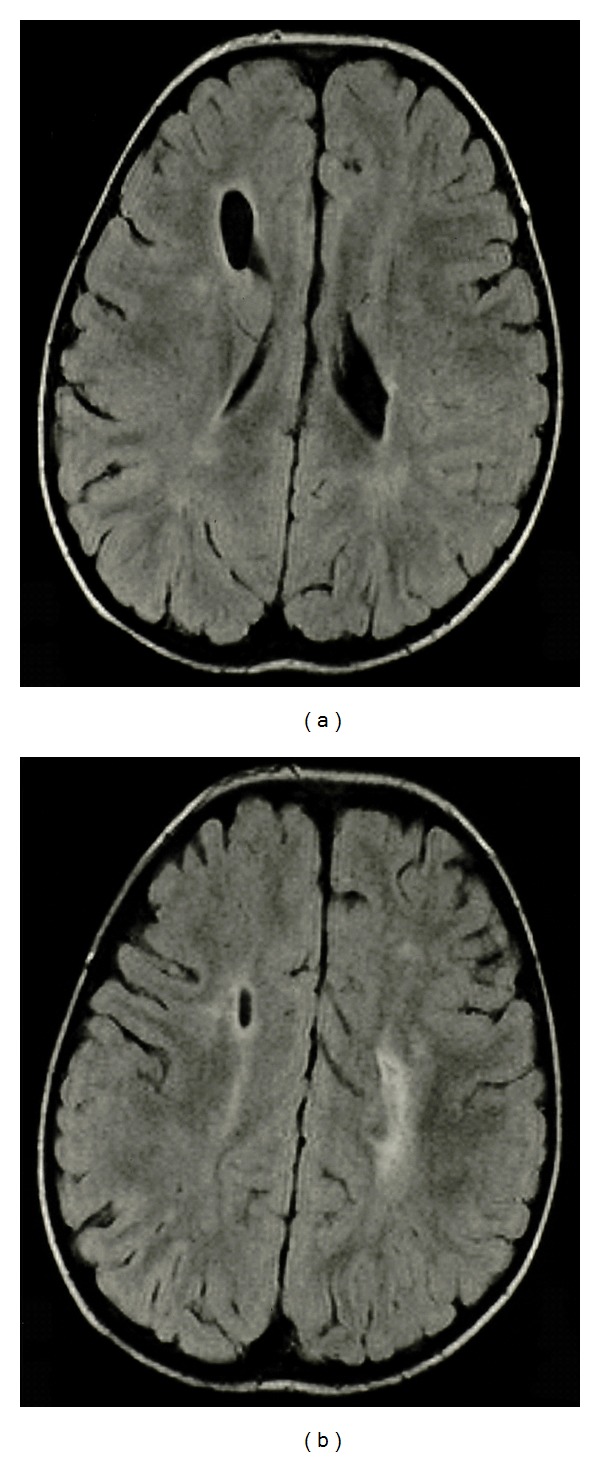
Transverse FLAIR MRI from the first case, family 2. Note irregular ventricular border in (a) and in a more rostral section, white matter hyperintense signal more prominent on the left in (b).

The second family includes two children with right hemiparetic cerebral palsy. The parents are not related. The mother has insulin-dependent diabetes mellitus and celiac disease while the father has autoimmune-related hypothyroidism. The first child is the product of a pregnancy complicated by preeclampsia in the last few weeks of gestation. An abscessed wisdom tooth prompted antibiotic use at approximately 30 weeks gestation. Alcohol or drug use was denied. This male child was born with a birth weight of 3000 grams (25–50th percentile) at 39 weeks gestation. Early developmental milestones were delayed, and he was found to have a left hand preference. Neurological examination showed mildly increased spastic hypertonia of the right upper extremity (Ashworth scale 1) and lower extremity (Ashworth scale +1) with no facial asymmetry and normal tone on the left. MRI demonstrated irregular dilatation in the region of the right frontal horn and left lateral ventricle with more prominent white matter hyperintense signal in the periventricular white matter on the left on both fluid attenuated inversion recovery (FLAIR) and T2-weighted MRI images (Figure 2). The spastic hypertonia of the right upper and lower extremities has been stable in keeping with the diagnosis of right hemiparetic cerebral palsy. He has otherwise been well but was recently diagnosed with insulin-dependent diabetes mellitus and celiac disease like his mother.

His sister also has right hemiparetic cerebral palsy. The pregnancy was uncomplicated with the exception of a mild urinary tract infection, the timing of which was not documented. During labor, however, maternal hypoglycemia prompted an emergent cesarean section. This female child was born at term with a birthweight of 4536 grams (>98th percentile). Bradycardia, mild breathing difficulties, and low normal glucose levels prompted admission to the special care nursery, but no intubation or active intervention was required. Early left hand preference prompted an MRI that demonstrated a left middle cerebral artery territory infarct. Her neurological examination shows spastic hypertonia of the right upper extremity (Ashworth scale +1) with little or no involvement of the lower extremity or face on that side.

Thrombophilia investigations in both children were normal with the exception of elevated activated protein C resistance 4.8 (adult reference range 1.9–4.5). However, the factor V Leiden DNA analysis was normal.

## 3. Discussion

While the aggregation of children with CP in families is uncommon accounting for less than 2% of cases, there is an estimated fivefold increase in the risk of recurrence in families with a child with CP [6]. In the mother of the first family, antithrombin III and protein S were found to be mildly low, while the Factor VIII, vonWillebrand antigen, and the anticardiolipin antibody were all elevated—all factors that may lead to a prothrombotic state. Notably, investigation of the children demonstrated no hereditary thrombophilias, and it was not until the mother suffered a hemorrhagic infarction that she was investigated. We hypothesize that all three children in the first family suffered *in utero* vascular events, possibly hemorrhagic infarction as was observed in the fifth pregnancy and in the mother. The presence of multiple prothrombotic factors in mom may have altered the fetoplacental circulation through the development of thrombi or intervillous hematomas (i.e., as was observed in the fifth pregnancy) leading to fetal complications. In all three children, asymmetric abnormalities were observed on MRI in keeping with the hypothesized vascular etiology [7] as well as variable combinations of ventriculomegaly, white matter hypoplasia, and periventricular leukomalacia. Vascular-mediated periventricular injury and selective vulnerability of oligodendroglia are a commonly reported mechanism in the preterm infant [8]. These same mechanisms may be responsible when ventriculomegaly is observed prenatally with static postnatal white matter hypoplasia [9]. Another possible explanation is an acquired maternal vasculitis resulting in hemorrhagic infarction, perhaps transmitted transplacentally to her progeny. However, no hereditable or autoimmune connective tissue disorders were identified in the family. The child of the fifth pregnancy died due to complications of complex congenital heart disease, and the cardiac malformation may also have contributed to the observed brain injury through the development of cardiac emboli or impaired fetal circulation. The surviving two children have exhibited a static encephalopathy over time, and imaging abnormalities have not progressed arguing against other disorders of hypomyelination or white matter hypoplasia [10].

In the second family, the mother developed a fever in both pregnancies—the first with an abscessed wisdom tooth at approximately 30 weeks gestation and the second with a urinary tract infection (gestation not documented). The neuroimaging findings in the first child demonstrated periventricular white matter involvement in keeping with an insult prior to 32 weeks gestation. The second child had cortical and white matter involvement in the distribution of the middle cerebral artery suggestive of a late gestation predominantly arterial vascular accident. In a population-based exploration of genetic susceptibility to CP, single nucleotide polymorphisms in genes associated with nitric oxide synthetase, endothelial protein C receptor precursor, and lymphotoxin *α* were associated with CP in term infants [11]. These genes are associated with the inflammatory response, coagulation, and function of the vascular endothelium. Polymorphisms of the interleukin-4 and interleukin-6 genes, which are components of the fetal inflammatory response, have also been implicated in the development of CP through increased susceptibility to infection [12, 13]. We hypothesize that the maternal infection, while benign and not directly infecting the fetus, may have triggered an inflammatory response which in the context of genetic susceptibility had less benign implications to the fetus. Moreover and similar to the first family, multiple potential risk factors for the development of CP were observed including maternal insulin-dependent diabetes and preeclampsia [14]. These multiple maternal factors may have converged in the second family significantly increasing the risk of subsequent pregnancies being complicated by an abnormal neurodevelopmental outcome. As with the first family, the children have not demonstrated any developmental regression or new neurological symptoms to suggest other neurodegenerative or neurometabolic disorders.

The convergence of multiple maternal and other possible unidentified genetic factors may in part explain the recurrence of CP in these two families. Given that the risk of having a second child with CP is already quite high as identified in the Swedish study [6], a thorough search for contributing factors is prudent in the overall assessment of recurrence risk. Ideally investigations would include examination of the placenta as a “record of the uterine environment” [15] and thrombophilia in both the mother and child [12]. Neuroimaging of the child and mother (if she has neurological symptoms) may provide additional insight into a suspected etiology and timing of injury [7]. The history of maternal infection, other autoimmune or inflammatory disorders, and obstetric factors like pre-eclampsia must also be documented [16]. Identification of these risk factors in both children and their mothers may prompt closer perinatal followup and should not be delayed in order to counsel parents prior to subsequent pregnancies.

In summary, our cases highlight the risk of recurrent CP in some families. We hypothesize that the presence of multiple environmental and hereditable factors converged in these two families resulting in an unusually high risk of recurrence. These observations underscore the importance of early identification and thorough investigation of children with cerebral palsy and their families. In the absence of a complete understanding of the causal pathway to CP, the clinician must be open to the possibility that unrecognized factors or the interaction of multiple factors may operate to disturb the intrauterine environment or development of the fetus. Evaluation of the placenta, MRI of the child and mother (if she has neurological symptoms), and examination of the mother and child for hereditary thrombophilias seem prudent suggestions. Our ability to counsel parents and families appropriately will depend on the depth of our search for known and yet unrecognized risk factors.

## Abbreviations

CP: Cerebral palsy
MRI: Magnetic resonance imaging.
